# Determinants of Tourism Stocks During the COVID-19: Evidence From the Deep Learning Models

**DOI:** 10.3389/fpubh.2021.675801

**Published:** 2021-04-09

**Authors:** Wen-Tsao Pan, Qiu-Yu Huang, Zi-Yin Yang, Fei-Yan Zhu, Yu-Ning Pang, Mei-Er Zhuang

**Affiliations:** School of Business, Guangdong University of Foreign Studies, Guangzhou, China

**Keywords:** COVID-19 era, deep learning, backpropagation neural network, quantum step fruit fly optimization algorithm, quantum particle swarm optimization algorithm, quantum genetic algorithm

## Abstract

This paper examines the determinants of tourism stock returns in China from October 25, 2018, to October 21, 2020, including the COVID-19 era. We propose four deep learning prediction models based on the Back Propagation Neural Network (BPNN): Quantum Swarm Intelligence Algorithms (QSIA), Quantum Step Fruit-Fly Optimization Algorithm (QSFOA), Quantum Particle Swarm Optimization Algorithm (QPSO) and Quantum Genetic Algorithm (QGA). Firstly, the rough dataset is used to reduce the dimension of the indices. Secondly, the number of neurons in the multilayer of BPNN is optimized by QSIA, QSFOA, QPSO, and QGA, respectively. Finally, the deep learning models are then used to establish prediction models with the best number of neurons under these three algorithms for the non-linear real stock returns. The results indicate that the QSFOA-BPNN model has the highest prediction accuracy among all models, and it is defined as the most effective feasible method. This evidence is robust to different sub-periods.

## Introduction

With the outbreak of the COVID-19, tourism suffered huge losses, and the stock prices of tourism were influenced dramatically. Simultaneously, the tourism industry's economic sustainability in the digital economy has been impacted by the epidemic as its growth has shifted from a steady increase to an unstable state. To better explore the tourism industry's economic sustainability and learn about the trend of tourism stock price, one must establish an excellent stock forecasting model. Stock price prediction has been regarded as an intractable task due to the stock market's intrinsic non-linearity and instability.

Since the end of 2019, the epidemic has impacted the economy and society, both in China and globally. China's National Bureau of Statistics announced a 6.8% year-on-year decline in real GDP for the first quarter of 2020. In terms of the micro-sector situation, China's tourism industry lost more than RMB 550 billion in revenue, equivalent to 2% of GDP in the first quarter, due to measures such as the travel ban imposed during the epidemic. With the epidemic under effective control in China, China's domestic tourism revenue during the May Day holiday in 2020 was RMB 47.56 billion, a recovery of 31.2% compared to the first quarter. In the digital economy, the tourism industry has always had good development opportunities. Still, the outbreak of the epidemic caused a short-term downturn in the tourism industry during this period. It can be seen from the above data that the economic sustainability of the tourism industry has been greatly impacted. As the tourism industry has been affected by the epidemic, stock prices in China's tourism industry are in a state of volatility. Also, they cannot be predicted accurately due to their stochastic features. The volatile stock price trends require better stock forecasting models to predict them.

In previous studies of stock prediction models, numerous scholars have pursued greater prediction accuracy. Kleijnen et al. (1) and Wang et al. (2) applied statistical analysis methods to predict stock price, including exponential smoothing method, linear regression method, moving average (MA) and autoregressive integrated moving average (ARIMA). Devi et al. (3) and Ariyo et al. (4) indicated that the ARIMA model is a suitable model to analyze time-series data. It is widely applied to analyze linear time series data. Nevertheless, Abu-Mostafa and Atiya (5) proposed that the stock price is non-linear and complex.

Some scholars combined a swarm intelligence algorithm with a stock prediction model to obtain higher prediction accuracy. For example, Zhang and Yan (6) constructed a single-step forward deep learning compound prediction model, the CEEMD-LSTM model, for the stock market based on the concept of “Decomposition-Reconstruction-Synthesis” and the deep learning prediction methodology. Many scholars invoked swarm intelligence algorithms to optimize stock prediction models' parameters to obtain higher prediction accuracy. For example, Wang and Zhuo (7) applied the FOA algorithm to optimize SVR parameters and combine them with support vector machines to develop a PCA-FOA-SVR stock price prediction model with high accuracy non-linear planning. However, compared with the QFOA algorithm, the FOA algorithm has a limited application range and a slightly lower parameter optimization accuracy. Bao et al. (8) adopted the GA algorithm to adjust the traditional LSTM model parameters, which outperformed the traditional LSTM model in predicting the data for CSI 500.

With artificial intelligence and science and technology development, more and more people pay attention to applying a deep learning model in the financial field. Nowadays, deep learning, especially backpropagation neural networks, has been widely used in data mining and prediction, which can effectively model non-linear statistical data. Wang et al. (2) proposed a hybrid approach combining ESM, ARIMA, and BPNN to utilize the most advantageous of all three models, and the weight of the proposed hybrid model (PHM) is determined by a genetic algorithm (GA). Shi et al. (9) proposed a hybrid method combining autoregressive and moving average (ARMA), backpropagation neural network (BPNN) and Markov model to forecast the stock price. Wu and Yong (10) used BPNN to predict the Shanghai Stock Exchange trend and pointed out that BPNN has its advantages in forecasting non-linear systems. Feng (11) adopted Levenberg-Marquardt (LM) algorithm for BPNN training to build a stock price prediction model. Sun et al. (12) proposed the Bayesian regularization method to optimize the training process of the BPNN to improve the generalization ability of the model and did empirical research about the closing price of Shanghai Stock. Huo et al. (13) established a three-layer BPNN. They utilized the LM-BP algorithm to forecast stock price, which has a faster convergence rate and overcomes the samples' redundancy and noise. Cao and Wang (14) constructed a stock price prediction study based on PCA and BP neural network algorithm. Yu et al. (15) used a local linear embedding dimensional reduction algorithm (LLE) to reduce the dimension of variables at first and then put the variables into BPNN to forecast stock price.

Although BPNN has achieved great success in stock price forecasting, there is still a question needed to solve how many nodes should be used in each layer. Despite abundant researches on the application of multilayer-BPNN, it's still a hard task to define the optimal architecture. If there are too few nodes in each layer, the architecture cannot learn from the data properly, whereas if there are too many nodes, it causes waste inefficiency of training the Neural Network. Thomas and Suhner (16). In general, there is no clear theoretical guidance for setting the number of nodes in each hidden layer. Some researches focused on this topic. For instance, Beigy and Meybodi (17) pointed out that the determination of the optimal topology of neural networks belongs to the class of non-deterministic polynomial-time (NP)-hard problems and proposed a survival algorithm to determine the number of hidden units of three layers neural networks. Thomas and Suhner (16) proposed a pruning approach to determine the optimal structure of neural networks. Khan et al. (18) conducted empirical research that indicated that BPNN for predicting stock prices with two hidden layers is more accurate than the sing layer and three and four hidden layers. However, Zhang and Shen (19) compared single hidden layer prediction models and the multiple hidden layers prediction model. The result showed that the three hidden layers' model has a better predictive ability than the former. Therefore, we consider that the number of hidden layers depends on the different data set and there is no absolutely optimal number of hidden layers.

Several papers in the literature examine the determinants of stocks during the COVID-19 era [see, e.g., (20–24)]. Still, several scholars seldom studied how to use the swarm intelligence algorithms to optimize the structure of BPNN for the pre-COVID era. However, these papers have not considered the NP-hard problems for the COVID-19 era. At this stage, the swarm intelligence algorithm is a good way to solve NP-hard problems. Therefore, a new Quantum Step Fruit Fly Optimization Algorithm (QSFOA) is proposed in this paper. This issue is the main contribution of our paper. Besides, quantum swarm intelligent algorithms (QSIAs), including QSFOA, QPSO and QGA, are respectively used to optimize the number of neurons in the multilayer-BPNN. The tourism industry's stock data is then applied to test the three models' predictive power as proof of the feasibility and superiority of the method proposed in the paper.

The structure of this paper is as follows. Section Research Method is to narrate the application of the relevant research methodologies in this paper. In section Empirical Analyses, the stock prediction model developed in this paper is measured by applying the stock data of two leading tourism companies in China. Besides, section Empirical Analyses introduces the comparison with the predictive ability of the QSFOA-BPNN model, QPSO-BPNN model and QGA-BPNN model. The findings of this paper and recommendations will be presented in section Conclusion.

## Research Method

Deep learning is a neural network with multiple hidden layers, whose concept is derived from artificial neural network research. It combines various neural network structures and is an extension of a Neural Network. And the neural network is a kind of machine learning technique that simulates the human brain to achieve artificial intelligence. This paper optimizes the deep learning models, multilayer-BPNN, using quantum swarm intelligence algorithms (QSIA) to design its network structure, including the number of neurons and the number of hidden layers. Therefore, some theories about BP Neural Networks and QSIA are introduced as follows.

### BP Neural Network

**Figure 1:**
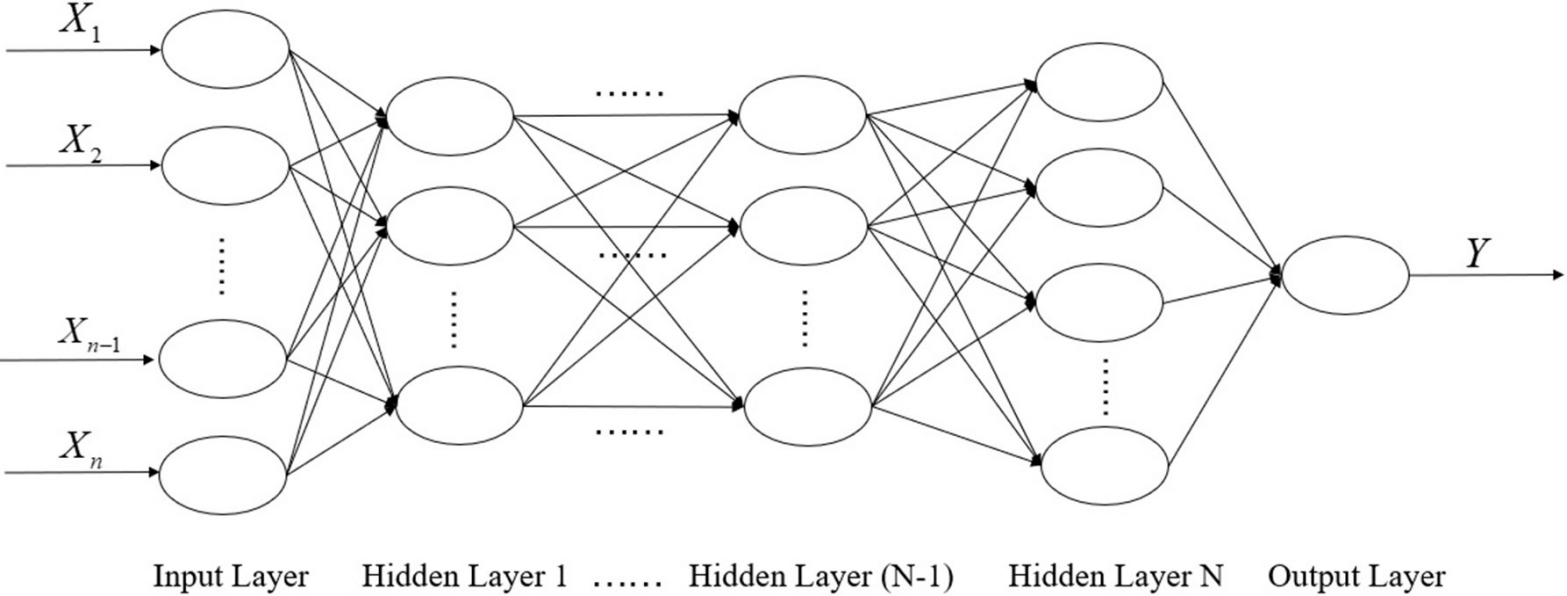
The structure of multilayer-BPNN.

BP neural network is one of the most commonly used artificial neural networks, which is also known as an error backpropagation neural network. BPNN is a kind of multilayer perceptron (MLP), and it is a feedforward network. It trains the network by error backpropagation algorithm. The training purpose of BPNN is to establish a non-linear mapping between input values *X* and output value *Y*. The weights and thresholds are adjusted continuously through error backpropagation, and finally, the error signal reaches the minimum. The training process of BPNN is mainly divided into two stages. The first stage is the forward propagation of the signal from the input layer to the hidden layer and finally to the output layer. The second stage is the backpropagation of errors, from the output layer to the hidden layer, and finally to the input layer, adjusting the hidden layer's weight and bias to the output layer to the hidden layers in turn. BPNN consists of three parts including input Layer, hidden layer and output layer. The structure of multilayer-BPNN is shown in Figure 1.

### QSIA

Normal swarm intelligence algorithms cannot deal with the shortcomings of premature convergence. Consequently, many scholars have applied quantum computing and other quantum principles to swarm intelligence algorithms to optimize better performance and faster convergence speed and algorithms such as Quantum Particle Swarm Optimization (QPSO), Quantum Genetic Algorithm (QGA).

This paper proposes a new Quantum Step Fruit Fly Optimization Algorithm (QSFOA) and compares its performance with QPSO and QGA. Thus, we will introduce basic knowledge about quantum computing at first. Secondly, we explain a few necessary steps and procedures about QSFOA in detail and then briefly introduce QPSO and QGA.

### Quantum Computing

#### Quantum bit boding

A quantum bit is a two-state quantum system in which a quantum bit is the smallest unit of information. A quantum bit is an inter-state between |1> and |0>. Namely, the different superposition states of |1> and |0>, so the state of a quantum bit can be indicated.

$$\begin{array}{l} |\psi >\;=\alpha |0>+\;\beta |1> \end{array}$$

In the above formula, |0> and |1> stand for the states of 0 and 1. α and β are satisfied by the following normalization conditions.

$$\begin{array}{l} {\left|\alpha \right|}^{2}+|\;\beta \;{|}^{2}=1 \end{array}$$

| α |^2^ and | β |^2^are the probability values between 0 and 1.

It can have seen that: if there is a system with m quantum bits, 2 m states enable to be represented simultaneously. It can be described as

$$\begin{array}{l} \left[\begin{array}{ccc} \alpha_{1} & \alpha_{2} & \cdots \;\alpha_{m}\\ \beta_{1} & \beta_{2} & \cdots \;\beta_{m} \end{array}\right] \end{array}$$

The above formula satisfies |α|^2^ + | β|^2^ = 1, i = 1, 2, ⋯ , m_°_

### Quantum Gate Update

In This article, the quantum rotating gate is chosen to update the probability amplitude before and after. The specific adjustment operation is as follows.

$$\begin{array}{l} U\left(\theta_{i}\right)=\left[\begin{array}{cc} cos\left(\theta_{i}\right) & -\text{sin}\left(\theta_{i}\right)\\ \text{sin}\left(\theta_{i}\right) & \text{cos}\left(\theta_{i}\right) \end{array}\right] \end{array}$$

Update the process as follows:

$$\begin{array}{l} \left[\begin{array}{c} \alpha_{i}^{{\;}^{\prime }}\\ \beta_{i}^{{\;}^{\prime }} \end{array}\right]=U\left(\theta_{i}\right)\left[\begin{array}{c} \alpha_{i}\\ \beta_{i} \end{array}\right]=\left[\begin{array}{cc} cos\left(\theta_{i}\right) & -\text{sin}\left(\theta_{i}\right)\\ \text{sin}\left(\theta_{i}\right) & \text{cos}\left(\theta_{i}\right) \end{array}\right]\left[\begin{array}{c} \alpha_{i}\\ \beta_{i} \end{array}\right] \end{array}$$

${\left(a_{i},\beta_{i}\right)}^{T}$ and ${\left(\alpha_{i}^{{\;}^{\prime }},\beta_{i}^{{\;}^{\prime }}\right)}^{T}$. The probability amplitude before and after the rotational gate update of the i-th quantum bit of the chromosome for the rotation angle is represented.

### QSFOA

The original FOA is a swarm intelligence optimization algorithm based on fruit fly foraging behavior proposed by Pan (25). Inspired by quantum computing, we propose a new Quantum Step Fruit Fly Optimization Algorithm (QSFOA) to address the shortcomings of FOA. The detailed procedure of QSFOA is as follows.

1. Randomly determine the initial population location.
  X_axis = 10^*^rand();
  Y_axis = 10^*^rand();
2. Randomly determine the initial quantum bits chromosome.
  $$\begin{array}{l} \;{\text{Q}}_{\text{q}}\left(t\right)=\left|\begin{array}{c} \alpha_{1}^{q}\\ \beta_{1}^{q} \end{array}|\begin{array}{c} \alpha_{2}^{q}\\ \beta_{2}^{q} \end{array}|\begin{array}{c} \alpha_{3}^{q}\\ \beta_{3}^{q} \end{array}|\begin{array}{c} \alpha_{4}^{q}\\ \beta_{4}^{q} \end{array}|\begin{array}{c} \alpha_{5}^{q}\\ \beta_{5}^{q} \end{array}|\begin{array}{c} \ldots \\ \ldots \end{array}|\begin{array}{c} \alpha_{p}^{q}\\ \beta_{p}^{q} \end{array}\right| \end{array}$$
3. Measure the qubit chromosome Q_q_(*t*) and obtain the binary coded chromosome.
  - Firstly, the measurement process will generate a matrix *QP*_*q*_(*t*).
    $$\begin{array}{l} \text{Q}{\text{P}}_{q}\left(t\right)=\left|r_{1}^{q}|r_{2}^{q}|r_{3}^{q}|r_{4}^{q}|r_{5}^{q}|\ldots |r_{p}^{q}\right| \end{array}$$
  - Secondly, “r” in the (2.1.2) is a random number between 0 and 1. When ${\left|a_{p}^{q}\right|}^{2}>r_{p}^{q}$, $p_{p}^{q}$ equals 0; otherwise, $p_{p}^{q}$ equals 1.
  - Thirdly, the state P_q_(*t*) is obtained after measurement comparison.
    $$\begin{array}{l} {\text{P}}_{q}\left(t\right)=\left|p_{1}^{q}|p_{2}^{q}|p_{3}^{q}|p_{4}^{q}|p_{5}^{q}|\ldots |p_{p}^{q}\right| \end{array}$$
  - Fourthly, by converting a binary code to a decimal number δ, the following result is decoded the following result.
    $$\begin{array}{l} \delta =\overset{10}{\underset{i=1}{\sum }}b_{i}\cdot 2^{\text{i}-1},X=U1+\frac{U_{2}-U_{1}}{2^{10}-1}x \end{array}$$
4. Random direction and distance to the fruit fly individual using smell to search for food.
  X(i) = X_axis+2^*^rand()-δ;
  Y(i) = Y_axis+2^*^rand()-δ;
5. Estimate the distance from the origin (Dist) because of the unknown food location, and then calculate the taste density determination value (S), which is the reciprocal of the distance.
  $$\begin{array}{l} \text{Dist}\left(\text{i}\right)=\sqrt{\text{X}{\left(\text{i}\right)}^{2}+\text{Y}{\left(\text{i}\right)}^{2}}\\ \text{ S}\left(\text{i}\right)=1/\text{Dist}\left(\text{i}\right) \end{array}$$
6. The smell concentration judgment value (S) is substituted into the smell concentration judgment function (or called Fitness function) and then figure out the smell concentration (Smell) of individual fruit flies in different locations.Smell(i) = objective function (S(i)) = RMSE
7. Identify the best concentration of flavor in this population of fruit flies.
  [bestSmell bestindex] = min(Smell);
8. Keep the best taste concentration value and the x, y coordinates. At this time, the fruit fly swarm will fly to this position by vision.
  X_axis = X(bestindex);
  Y_axis = Y(bestindex);
  Smellbest = bestSmell;
  If failure to find the minimum of root mean square error (RMSE), a quantum rotating gate is used to change Q_q_(*t*).
9. Iteratively find the optimal value until minimum RMSE is found. Steps 3–6 need to be repeated and determined whether the flavor concentration is superior to the previous iteration of flavor concentration, and if so, step 7.

### QPSO

Sun et al. (26) proposed a particle swarm optimization with quantum behavior with few parameters and a simple equation.

The QPSO, to increase the randomness of particle position, cancel the particle's movement direction property, using quantum mechanics, makes each particle have quantum behavior. The specific steps are as follows.

1. Initialize the locations of particles in space. The position vector of the particle is
  $$\begin{array}{l} x_{i}\left(t\right)=\left(x_{i1}\left(t\right),x_{i2}\left(t\right),\cdots \;,x_{iD}\left(t\right)\right),i=1,2,\cdots \;,M;\\ \;pbest_{i}=x_{i} \end{array}$$
  In the above formula, *pbest*_*i*_ is the optimal position of individual particles.
2. Calculate the value of fitness function on each particle and select the particle with the best value to be globally optimal particle *gbest*;
3. Determine each particle's local factor as *p*_*i*_;
  $$\begin{array}{l} p_{i}=\varphi \times pbest_{i}+\left(1-\varphi \right)\times gbest\\ \varphi =\frac{\phi_{1}}{\phi } \end{array}$$
4. Update *Mbest*(mean particle best position);
  $$\begin{array}{l} \text{Mbest}=\frac{1}{M}\overset{m}{\underset{i=1}{\sum }}pbest_{i} \end{array}$$
  In the above formula, M represents the size of the particle group, and the average value of is represented by *Mbest*.
5. Modify the search range of particles, which is *L*_*i*_(*t*);
  $$\begin{array}{l} L_{i}\left(t\right)=2\beta \cdot \left|Mbest-x_{i}\left(t\right)\right| \end{array}$$
  β is Contraction-Expansion coefficient, controlling convergence speed.
6. Particles undergo a new evolution;
  $$\begin{array}{l} x_{ij}\left(t\right)=p_{ij}\left(t\right)\pm \beta \left|Mbest-x_{ij}\left(t\right)\right|\cdot \text{ln}\left(\frac{1}{u_{ij}\left(t\right)}\right) \end{array}$$
7. Calculate a current fitness value of the particles, comparing it with the individually optimal fitness value that it has gone through, then designate the particle with the highest adaptation as the new *pbest*_*i*_;
8. All of particles' current adaptation value is compared to the globally optimal fitness value in the population, and the particle with the highest adaptation is designated as *gbest*;
9. Repeat steps 2–8 until the minimum RMSE is reached, then it is terminated.

### QGA

Han and Kim (27) proposed a new quantum genetic algorithm based on quantum chromosome coding. The key to the new genetic algorithm is the introduction of quantum revolving gates to the parent generation. The specific process of quantum genetics used in this paper is as follows.

1. An initial population is first performed to randomly generate *n* chromosomes encoded in quantum bits.
2. Take one measurement for each individual in the initial population to obtain the corresponding deterministic solution.
3. Perform an adaptation assessment for each deterministic solution and record the optimal individual and their fitness value.
4. They are determining whether the conditions for termination have been met.
5. Measurement of all individuals in the population and their fitness value to defined values.
6. Apply adjustments to individuals using quantum revolving gates to obtain new populations.
7. Recording the optimal individual and the corresponding fitness value.
8. Increase the number of iterations, and continue with step (4) until the smallest RMSE is found.

## Empirical Analyses

### Sample Data and Variables

After finding the price and total value of numerous tourism stocks and other relevant information through Eastmoney, this paper compares the data and preliminarily identifies ten enterprises such as China Tourism Group Duty-Free Corporation Limited, and further randomly selects two leading enterprises, Utour Group Co., Ltd. and China Tourism Group Duty-Free Corporation Limited. Therefore, this paper collects these two enterprises' daily stock information from the Wind database from October 25, 2018, to October 21, 2020, with 483 observations. We first import all the data from 16 indexes of the two tourism enterprises' stocks into the R language, then discretize the data using the “discretization” function in the rough set calculation method. Finally, the function “FS.all.reducts.computation” is used to reduce the indexes. After eliminating the useless indexes through a rough set, the 16 technical indexes such as opening price are reduced to 12 technical indexes such as opening price, highest price, lowest price, turnover (million RMB) and RSI as the independent variables in this paper.

**Figure 2:**
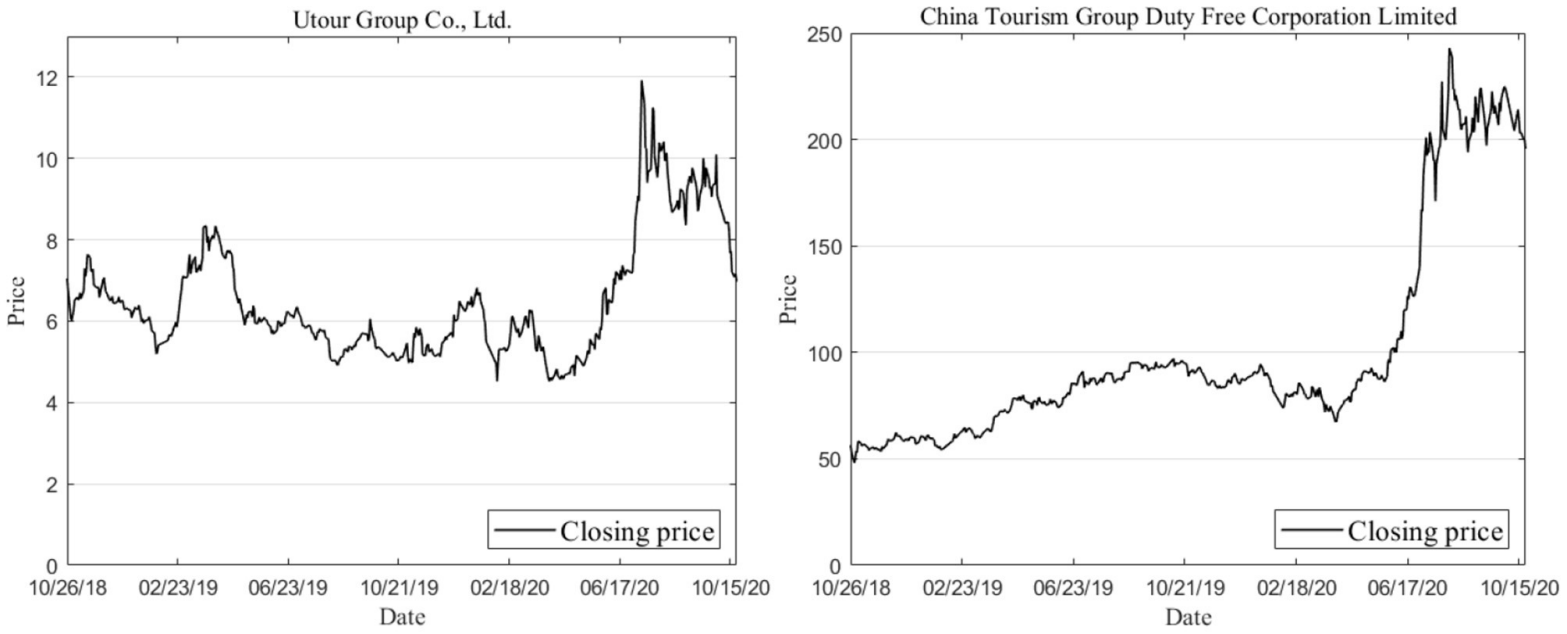
Movement of closing prices of two enterprises.

In this paper, MATLAB R2019a software is used for the analysis. Eighty percent of entire data sets (if it's not an integer, round it off) are used as training data to construct the model. The rest of the 20% data are then used as test data to perform the prediction accuracy analysis of the QSFOA-BPNN, QPSO-BPNN and QGA-BPNN. The basic information on the two enterprises' technical indexes can be seen in Tables 1, 2. Furthermore, the price movement chart of the two companies can be seen in Figure 2.

**Table 1:** The descriptive statistical value of technical indexes for Utour Group Co., Ltd.

	***N***	**Minimum**	**Maximum**	**Mean**	**SD**	**Variance**
Opening price (¥)	483	4.46	12.05	6.50	1.45	2.11
Highest price (¥)	483	4.60	12.92	6.67	1.53	2.35
Lowest price (¥)	483	4.46	10.92	6.36	1.37	1.89
Turnover (million)	483	6.91	1358.47	128.06	148.85	22157.21
Volume	483	1396500	118807800	17762687	15730330	247443289300921
MA (10)	483	25.06	44.35	37.67	5.69	32.39
MA (20)	483	25.02	43.79	37.49	5.67	32.13
MA (60)	483	24.75	42.15	36.79	5.71	32.56
MA (120)	483	24.47	41.81	35.74	6.06	36.76
MACD	483	−2.41	3.08	0.25	0.96	0.92
TECH_DIFF	483	−2.41	3.08	0.25	0.96	0.92
RSI	483	7.12	99.22	55.98	22.86	522.80
Closing price (¥)	483	4.52	11.92	6.52	1.46	2.13

**Table 2:** The descriptive statistical value of technical indexes for China tourism group duty-free corporation limited.

	***N***	**Minimum**	**Maximum**	**Mean**	**SD**	**Variance**
Opening price (¥)	483	47.96	246.99	99.30	48.72	2373.19
Highest price (¥)	483	50.85	249.00	101.59	50.66	2566.88
Lowest price (¥)	483	47.90	226.00	97.40	47.05	2213.89
Turnover (million)	483	236.84	9194.45	1302.75	1332.35	1775148.81
Volume	483	3163100	46052300	11861732	6551572	42923104422383
KDJ(D)	483	16.05	94.01	56.02	18.68	348.78
KDJ(J)	483	−19.12	117.18	55.82	34.66	1200.98
MA(10)	483	53.66	223.33	98.22	47.70	2275.11
MA(20)	483	54.67	215.73	96.65	46.05	2120.69
MA(60)	483	56.82	212.13	90.46	37.52	1407.51
MA(120)	483	58.92	168.14	83.33	23.74	563.65
RSI	483	10.95	95.81	55.31	17.05	290.84
Closing price (¥)	483	48.12	243.00	99.58	48.82	2383.79

It can be observed from Figure 2 that a trend of constant fluctuation existed in the closing price levels of the two enterprises from October 2018 to April 2019. At the beginning of the epidemic, Utour Group Co., Ltd.'s closing price showed a small upward trend, but the overall trend was still downward. China Tourism Group Duty-Free Corporation Limited's closing price also showed a downward trend. However, after April 2020, the two enterprises' closing prices have risen rapidly and have maintained a relatively high level.

Since the architecture of BPNN will influence the predictive ability of BPNN, while existing methods cannot enable BPNN to get a great predictive ability, it shall be studied. In this paper, quantum swarm intelligence algorithms (QSIA) are used to determine the number of neurons in each hidden layer independent. In the meantime, the dependent variable is the root mean square error (RMSE). The RMSE is the objective function in these three QSIA, and the QSIA is aimed to find the minimum of RMSE corresponding to each structure of BPNN.

**Figure 3:**
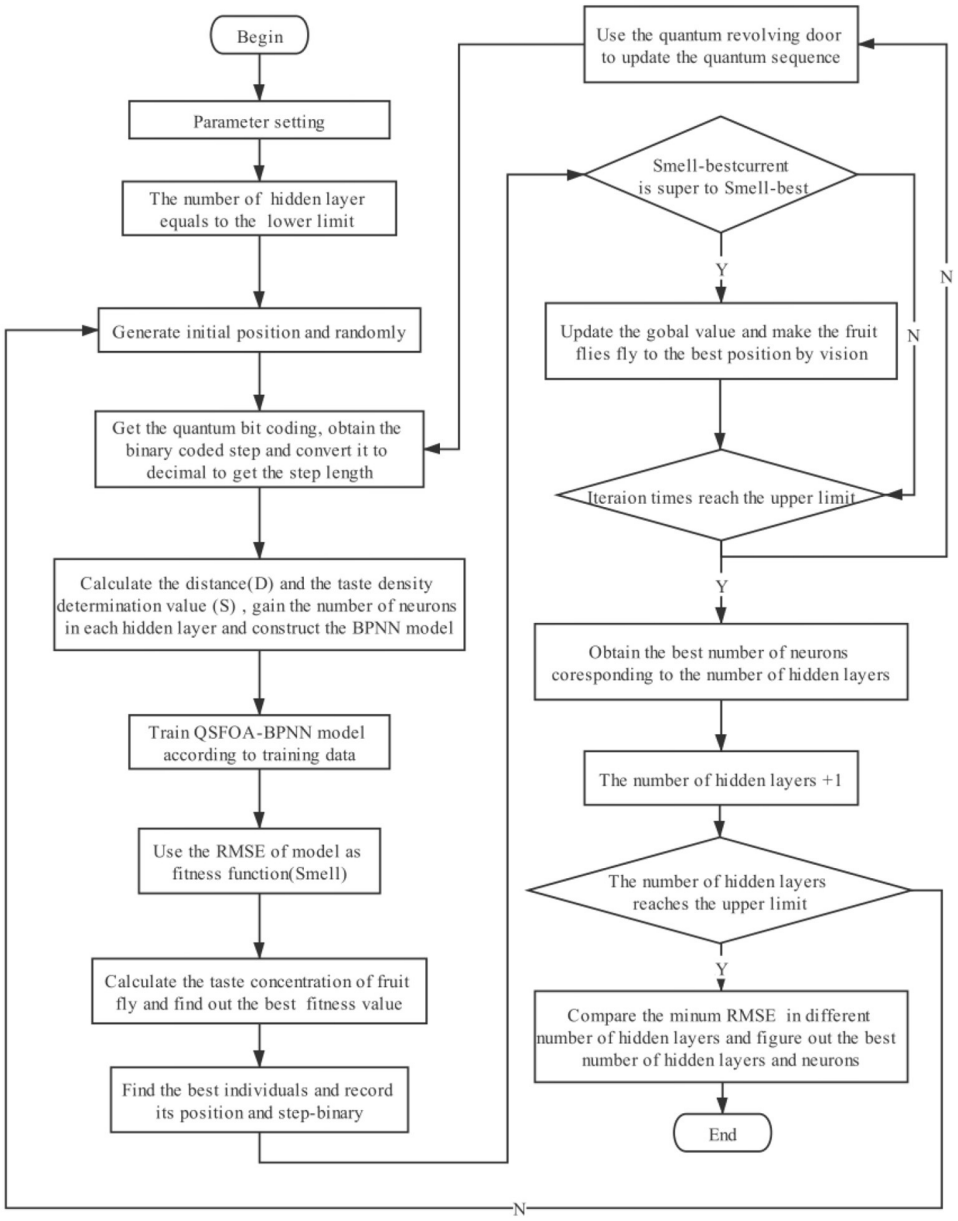
Specific algorithm flow chart of QSFOA-BPNN.

The way to use QSFOA to optimize BPNN is to calculate the distance between the fruit flies' location and the origin coordinate (0, 0). Then, we calculate the reciprocal that is smell concentration judgment value (S). If it's not an integer, we need to round it off, which refers to the number of neurons in each hidden layer. The number is substituted into the BPNN and train BPNN through the data set and then record the RMSE. The specific algorithm flow chart of QSFOA-BPNN is shown in Figure 3.

In our research, the difference between the prediction result of the QSFOA-BPNN, QPSO-BPNN, and QGA-BPNN is calculated firstly to be used as an error term. In the BP neural network, we adopt MATLAB to a self-edit program to perform the error term's prediction. The parameter setting values of BPNN include iterative number of 100, train goal of 10^−5^, learning rate of 0.1. The input layer has 12 nodes (that is, 12 input variables which refer to 12 technical indexes), and the output layer has one node (that is, one output variable which refers to the closing price).

Concerning BPNN with multiple hidden layers, there is no clear theoretical guidance for setting the number of each hidden layer. To figure out the best architecture of multilayer-BPNN, we set up the number of layers in BPNN as *L* ∈ {3, 4, 5}. According to Zurada (28), in this paper, we adopted the following formula to determine the number of neurons in each hidden layer.

$$\begin{array}{l} h_{i}=\sqrt{m+1}+\alpha \end{array}$$

Where *i* ∈ {1, 2, …, *L*}, *m* denotes the neurons count of the input layer, and [0, 10]

Since there are 12 variables taken as an input vector, the input layer consists of twelve neurons. This definition is *m* = 12. Meanwhile, the following day's closing price is taken as the output, so we have *n* = 1.

Based on the equation, it can be found that 4 ≤ *h* ≤ 14. Therefore, we fetch *h* in the set of {4, 5, …, 14}. It can be seen from the above equations that with increasing the value of *m*, the suitable range of neurons in hidden layers should be enlarged. Therefore, the upper of neurons in each hidden layer is 14, and the lower limit is four.

Then, concerning the initial parameter set up of QSFOA, the random initialization fruit fly swarm location range is [−10, 10], the quantum fly step of iterative fruit fly food searching is [−5, 5], the iteration number is 100, fruit fly population is 5, the length of step-binary coding is 10. Concerning the setting of QPSO, the iteration number is 100, and the particle population is 5. About the setting of QGA, the iteration number is 100, the population is five, and the length of binary coding is 10.

In the different number of hidden layers, we calculate RMSE separately, record the number of neurons corresponding to the minimum of RMSE, and finally compare the RMSE under each hidden layer to find out the number of hidden layers and neurons corresponding to the minimum RMSE, to determine the best neural network structure.

### General Comparison of the Prediction Capabilities of Three Models

**Figure 4:**
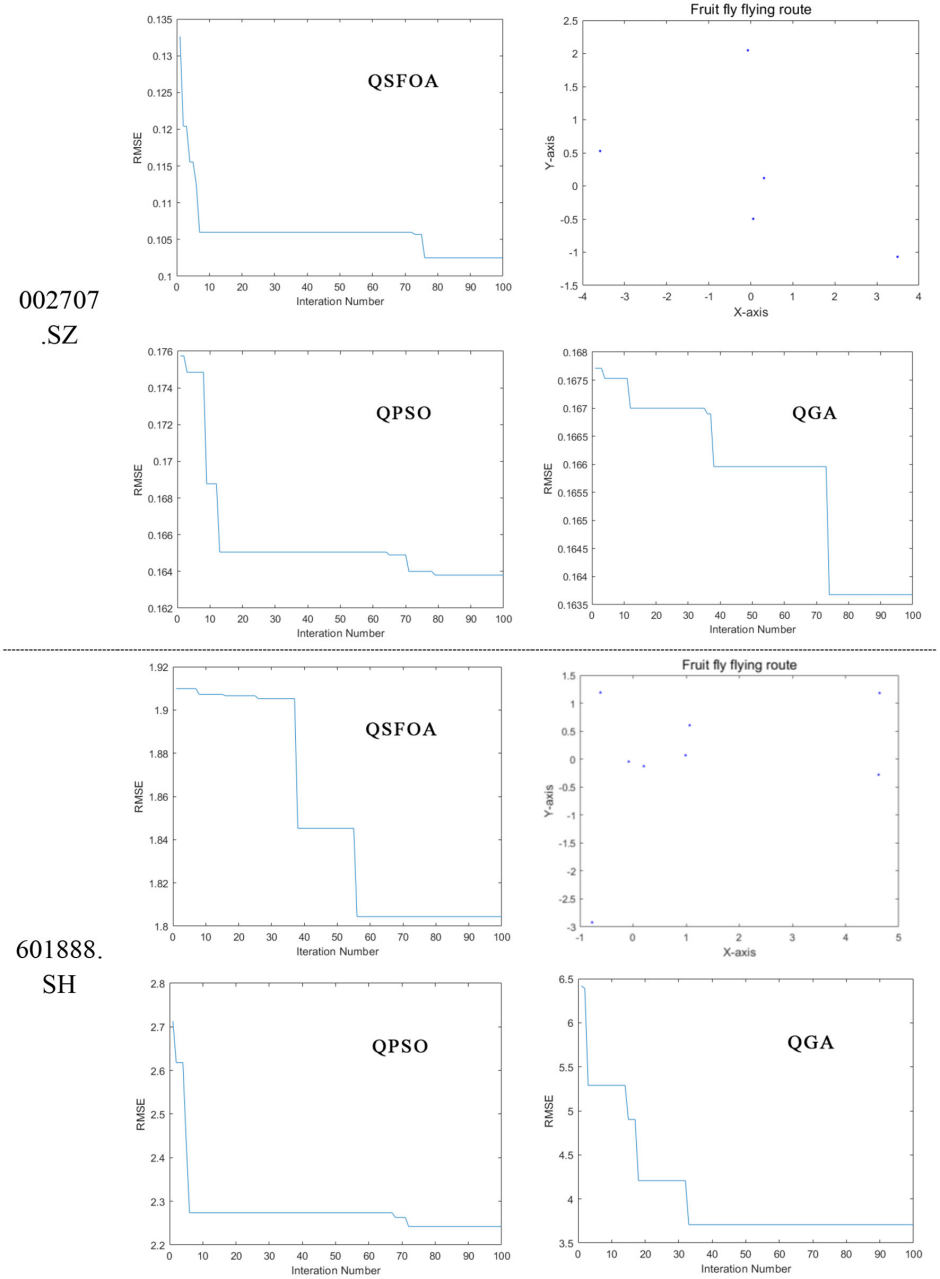
Trend chart of RMSE's iterative decline and fruit fly flying route.

All of the algorithms are run five times independently, and the best results obtained from runs are saved. The final results of the test data are shown in Table 3, and the best structure is shown as a vector that (*m, h*_1_*, h*_2_*, …, h*_*L*_*, n*) where *m* denotes the neurons count of the input layer and *L* denotes the number of hidden layers and *h*_*i*_ (*i* = 1, 2, …, *L*) denotes the *i*th hidden layer. Figure 4 shows the trend chart of RMSE's iterative decline under the best results in times running, and it also shows the fruit fly flying route of QSFOA while optimizing the BPNN.

**Table 3:** Final prediction results of three models.

**Stock**	**Model**	**CE**	**RMSE**	**MAPE**	**MAD**	**Best structure**
002707.SZ	QSFOA-BPNN	0.9850	0.1025	1.2416	0.0772	12,11,9,14,1
	QPSO-BPNN	0.9773	0.1639	1.4314	0.0978	12,14,9,10,1
	QGA-BPNN	0.9782	0.1637	1.0749	0.0879	12,11,6,8,11,1
601888.SH	QSFOA-BPNN	0.9814	1.8054	1.0137	1.0127	12,10,7,12,1
	QPSO-BPNN	0.9791	2.2620	0.7659	0.8578	12,8,9,10,6,1
	QGA-BPNN	0.9664	3.7008	1.1512	1.5050	12,9,6,4,1

Four evaluation indexes are used to compare the forecasting ability of three models, and the formula of four indexes separately is:

Coefficient of efficiency (CE), whose formula is:

$$\begin{array}{l} CE=1-\frac{\sum {\left(X_{t}-{\hat{X}}_{t}\right)}^{2}}{\sum {\left(X_{t}-{\bar{X}}_{t}\right)}^{2}} \end{array}$$

Root Mean Squared Error (RMSE), whose formula is:

$$\begin{array}{l} RMSE=\sqrt{\frac{\sum {\left(y_{i}-{\hat{y}}_{i}\right)}^{2}}{n}} \end{array}$$

Mean Absolute Percentage Error (MAPE), whose formula is:

$$\begin{array}{l} MAPE=\frac{1}{n}\overset{1}{\underset{n}{\sum }}\frac{\left|y^{{\;}^{\prime }}-y\right|}{y} \end{array}$$

Median Absolute Deviation (MAD), whose formula is:

$$\begin{array}{l} MAD=\frac{1}{n}\overset{n}{\underset{i=1}{\sum }}\left|X_{i}-m\left(x\right)\right| \end{array}$$

Among the four evaluation indexes, the closer the first index(*CE*) to one, the accurate the model. On the contrary, the closer the second to fourth index to zero, the accurate the model. Besides, the best structure optimized by these three QSIA about two companies is shown in the fifth index, and the last one is the running time of different algorithms.

We can find from Table 3 that QSFOA-BPNN model evaluation indexes in the first group of data are as follows, MAPE is 1.2416, which is not much different from the other two models. While CE is 0.9850, RMSE is 0.1025, and MAD is 0.0772, all of which are better than the other two models. Therefore, the prediction performance of stock 002707.SZ gives a ranking result about the three models of QSFOA-BPNN > QGA-BPNN > QPSO-BPNN. The QSFOA-BPNN model has a MAPE of 1.2416 and a MAD of 1.0127, which is in the middle of the three models from the secondary data set. While the CE is 0.9814 and the RMSE is 1.8054, the QSFOA-BPNN model has the best goodness of fit and the lowest error compared to the remaining two models. The prediction performance of stock 601888.SH shows that QSFOA-BPNN > QPSO-BPNN > QGA-BPNN.

**Figure 5:**
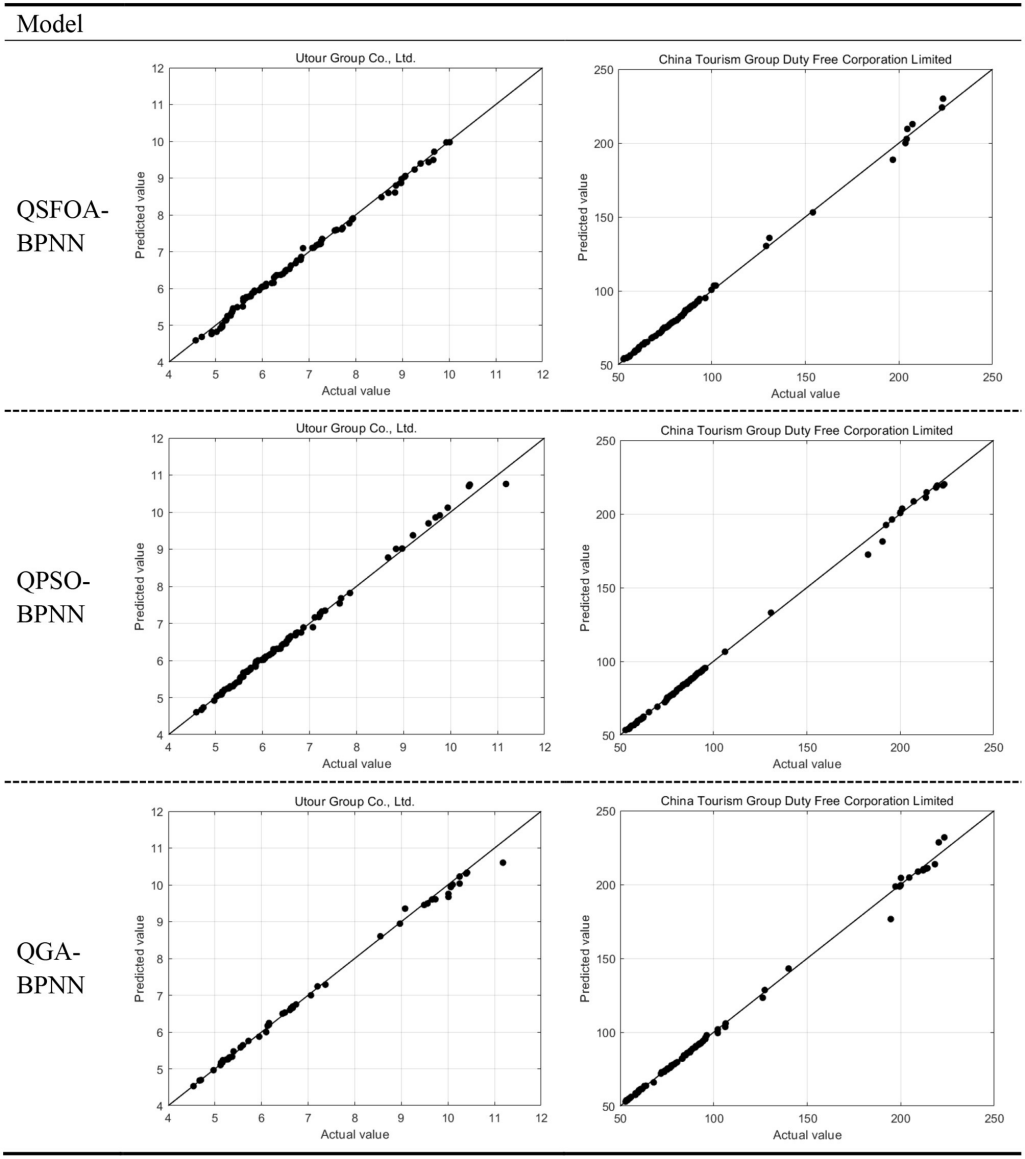
The sample points clustering trend chart of the prediction results.

The upper half part of Figure 5 shows the trend chart, where the three algorithms in sample data from 002707.SZ presents a continuous decrease in RMSE between calculated predictive value and target value during the optimization process. The research results show that RMSE between the predictive value and target value is 0.1025 after iterative optimization of the number of BPNN neurons by QSFOA; that is 0.1639 after iterative optimization of the number of BPNN neurons by QPSO; that is 0.1637 after iterative optimization of the number of BPNN neurons by QGA, so it demonstrates that QSFOA has the best ability in terms of BPNN optimization.

The lower half of Figure 5 shows the trend chart, where the three algorithms in sample data from 601888.SH presents a continuous decrease in RMSE between calculated predictive value and target value during the optimization process. The research results show that RMSE between the predictive value and target value is 1.8054 after iterative optimization of the number of BPNN neurons by QSFOA; that is 2.2720 after iterative optimization of the number of BPNN neurons by QPSO; that is 3.7008 after iterative optimization of the number of BPNN neurons by QGA, so it demonstrates that QSFOA has the best ability in terms of BPNN optimization.

The fruit fly flying routes in the two stock models reveal that all the fruit flies in the QSFOA-BPNN model have very random flight paths, making fruit flies easily jump out of the solution of local extreme value and find the solution of the global optimum. Therefore, QSFOA has a high optimization capability.

In our research, we analyze that some of the algorithms may be stuck at local minima, and they cannot find the best nodes number of the current structure of hidden layers. Simultaneously, it has reached the iteration time, which may be why the best structure under different QSIA has a different number of hidden numbers.

All of the algorithms are implemented in an Intel Core (TM) i5-8300H CPU 2.30 GHz processor, 8.00 GB DDRIII of RAM. Furthermore, this article uses the sample data to test three prediction models for two stock closing prices five times. The test results are shown in Table 4. We can learn from Table 4 that QSFOA performs best in the running time because it reduces calculating complexity with high calculating speed in general.

**Table 4:** Average running time(s) of three models that have been tested for five times.

**Algorithm**		**QSFOA-BPNN**	**QPSO-BPNN**	**QGA-BPNN**
002707.SZ	Running time (s)	1428.1995	1929.3138	2028.0438
601888.SH	Running time (s)	965.2677	1384.6192	1069.0291

Figure 5 shows the prediction results of the test data in QSFOA-BPNN, QPSO-BPNN and QGA-BPNN. The horizontal axis corresponds to the stock's closing price of actual value, while the vertical axis is the closing price of the predicted value. The closer the sampling point is to the diagonal, the more accurate the prediction results will be. According to the samples' clustering trend, we can see that the three models have a good predictive ability. However, the model optimized by QSFOA can predict the closing price of the two tourism companies more accurately than the model optimized by QGA and QPSO.

## Conclusion

Based on the information trend of tourism in the digital age, this paper takes the closing prices of tourism stocks and observes their trends to develop a prediction model for tourism stocks with better prediction accuracy. We explore the future economic sustainability of tourism driven by the digital age covering the COVID-19 era. We establish the deep learning models for forecasting, considering that a better structure of BPNN enables us to get better prediction results. For this purpose, this paper adopts the quantum swarm intelligence algorithms (QSIA), including the QSFOA, the QPSO and the QGA, associated with the BPNN adjusted with the number of neurons hidden layer independent variable accuracy analysis for the prediction of the closing price of the stocks. The QSFOA-BPNN model results show that the Utour Group Co., Ltd and China Tourism Group Duty-Free Corporation Limited performs more accurately with the QPSO-BPNN and the QGA-BPNN approaches. It is important to note that our models do not apply to the large volume of data to validate the prediction capability. Therefore, the QSFOA-BPNN model optimized by the QSFOA algorithm can be used as a research direction for future papers.

## Data Availability Statement

The raw data supporting the conclusions of this article will be made available by the authors, without undue reservation.

## Author Contributions

W-TP, Z-YY, and Y-NP: wrote the paper. Q-YH: data collection and wrote the paper. F-YZ: methodology and software. M-EZ: funding access and reviewed the paper. All authors contributed to the article and approved the submitted version.

## Conflict of Interest

The authors declare that the research was conducted in the absence of any commercial or financial relationships that could be construed as a potential conflict of interest.
